# Metagenome-assembled microvirus and cressdnavirus genomes from fecal samples of house mice (*Mus musculus*)

**DOI:** 10.1128/mra.00331-24

**Published:** 2024-07-08

**Authors:** Elise N. Paietta, Simona Kraberger, Joy M. Custer, Karla L. Vargas, Erin Ehmke, Anne D. Yoder, Arvind Varsani

**Affiliations:** 1 Department of Biology, Duke University, Durham, North Carolina, USA; 2 The Biodesign Center for Fundamental and Applied Microbiomics, Center for Evolution and Medicine, School of Life Sciences, Arizona State University, Tempe, Arizona, USA; 3 Duke Lemur Center, Durham, North Carolina, USA; 4 Structural Biology Research Unit, Department of Integrative Biomedical Sciences, University of Cape Town, Cape Town, South Africa; Katholieke Universiteit Leuven, Leuven, Belgium

**Keywords:** microvirus, cressdnavirus, *Mus musculus*

## Abstract

House mice, *Mus musculus*, are highly adapted to anthropogenic spaces. Fecal samples were collected from house mice entering primate enclosure areas at the Duke Lemur Center (Durham, NC, USA). We identified 14 cressdnavirus and 59 microvirus genomes in these mouse feces.

## ANNOUNCEMENT

Invasive rodents alter ecosystem dynamics by outcompeting endemic wildlife for resources and frequently moving between wild and human-inhabited spaces (1 – 5). House mice (*Mus musculus*), in particular, have become one of the most widespread invasive species, being transported across the planet through human movement and adapting remarkably well to human-altered landscapes (6).

As part of a larger study sampling lemurs, humans, and rodents at the Duke Lemur Center (Durham, NC, USA) under IACUC #A161-21-08, fecal samples were collected in August 2021 from two *M. musculus* trapped indoors in lemur enclosure areas. Fecal samples were frozen at −80°C until extraction. Once thawed, fecal samples were homogenized with SM buffer [0.1 M NaCl, 50 mM Tris-HCl (pH 7.4)] and centrifuged briefly at 8,000 rpm. Using the Roche High Pure Viral Nucleic Acid Kit (Roche Diagnostics, Germany), DNA was extracted from 200 µL of each supernatant. DNA extract was amplified using the Templiphi kit (GE Healthcare, USA). After library preparation with the Illumina DNA Prep Kit, libraries were sequenced on an Illumina NovaSeq 6000 at the Duke Center for Genomic and Computational Biology yielding 18,080,238 and 23,175,508 paired reads for each of the two samples. Paired-end reads were trimmed using Trimmomatic v0.39 (7) and assembled with MEGAHIT v.1.2.9 (8). Circular contigs were identified by terminal redundancy based on a >10 nt repeat. Diamond v2.1.9 (9) BLASTx was used to identify viral-like sequences against a local NCBI RefSeq viral protein database (release 220). Cenote Taker2 v2.1.5 (10) and VIBRANT v1.2.1 (11) were used to annotate viral genomes. Viral genomes with >98% identity were clustered into virus operational taxonomic units (vOTUs) with SDT v1.2 (12) and used as a reference to map reads with BBMap v38.12 (13). Web-based BLASTn was used to determine the similarity of viruses characterized in this study to known virus genomes. All bioinformatics tools were used with default settings.

**Fig 1:**
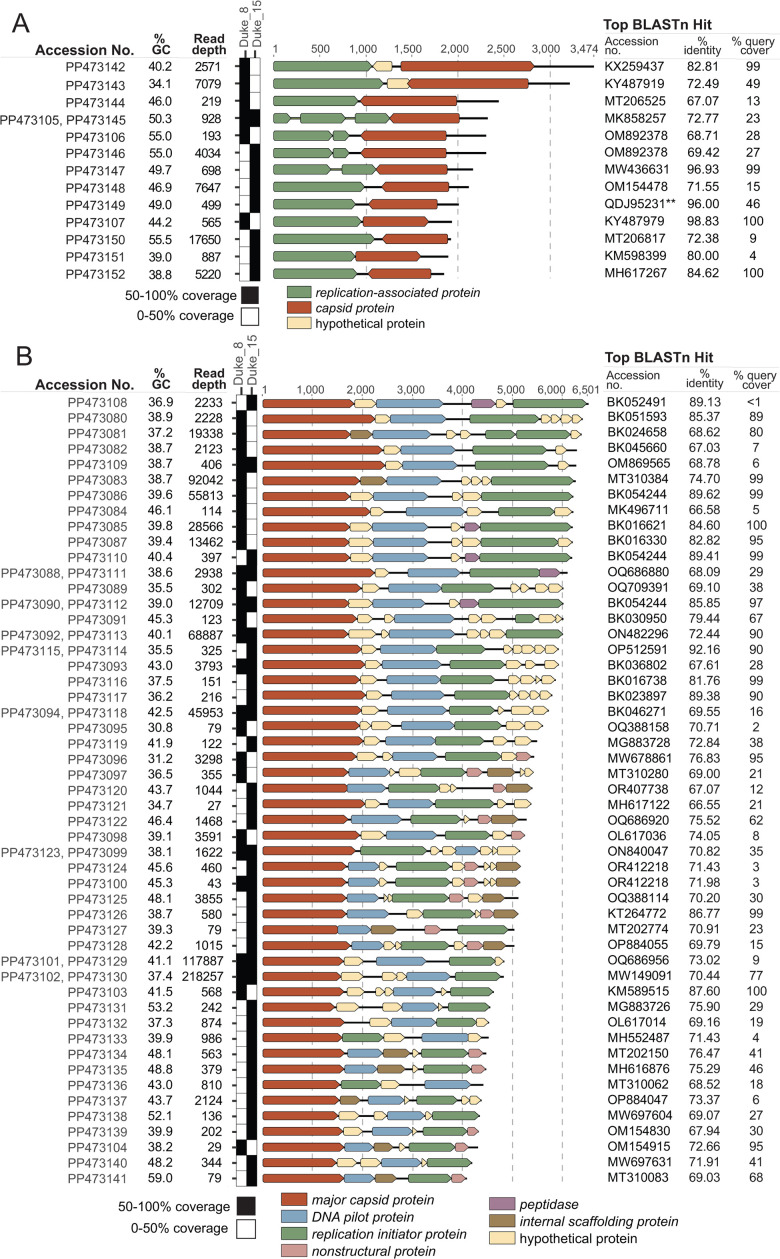
(A). Genome organizations of the cressdnaviruses were identified in *M. musculus* feces in this study. A summary of the accession numbers, GC content, read depth, and top BLASTn hit for each distinct genome is presented. **This BLAST hit represents a Rep protein BLASTp instead of a full genome comparison for PP473149.** (B)** Genome organizations of the microviruses identified in *M. musculus* feces in this study. A summary of the accession numbers, GC content, read depth, and top BLASTn hit for each distinct genome is presented. Genomes characterized in this study with >98% nt identity are represented by one row. For (A) and (B), genome coverage plots based on vOTU read mapping depict the presence of all vOTUs across Duke_8 and Duke_15. Black squares represent 50%–100% genome coverage and serve as a high-confidence proxy of vOTU presence.

We identified 14 cressdnavirus and 59 microvirus genomes from feces collected from two *M. musculus* individuals (Duke_8, Duke_15). The *Cressdnaviricota* phylum is composed of single-stranded DNA viruses infecting an array of eukaryotic hosts including plants, animals, fungi, and protists (14). The 14 cressdnavirus genomes encode a capsid and replication-associated protein, range in length from 1,847 to 3,474 nt, and GC content of 34.1%–55.0% (Fig. 1A). PP473106 and PP473146 share 97.5% similarity and fall within the *Smacoviridae* family, a group predicted to infect gut archaea (15). PP473147 is a member of the *Genomoviridae* family, a group of likely fungi-infecting cressdnaviruses (16). The unclassified cressdnavirus genomes (*n* = 11) identified in this study share similarities with viruses identified from water (*n* = 5), soil (*n* = 1), fish tissue (*n* = 1), damselflies (*n* = 1), capybara feces (*n* = 1), and tortoise feces (*n* = 2).

Microviruses are small, single-stranded DNA bacteriophages (17). The 59 microvirus genomes identified encode at least a major capsid protein, DNA pilot protein, and replication initiator protein, range in length from 4,066 to 6,501 nt, and range in GC content from 30.8%–59.0% (Fig. 1B). The distinct (<98% similarity) microvirus genomes (*n* = 51) share the highest similarity with microviruses characterized from rodent (*M. musculus, Dipodomys merriami, Rattus norvegicus,* and *Sigmodon arizonae*) feces and tissue (*n* = 8), human samples (*n* = 17), water (*n* = 9), ungulate feces (*n* = 5), soil (*n* = 3), fish tissue (*n* = 2), bat feces (*n* = 2), insects (*n* = 2), reptile feces (*n* = 1), avian feces (*n* = 1), dog feces (*n* = 1), and airborne particulate (*n* = 1). The identified microviruses likely infect enterobacteria within *M. musculus*.

## Data Availability

The sequences of cressdnaviruses and microviruses in this study have been deposited in the NCBI SRA under SRR28214738 and SRR28214739 and GenBank accession numbers PP473080–PP473152.
